# The biogenesis and maintenance of PSII: Recent advances and current challenges

**DOI:** 10.1093/plcell/koae082

**Published:** 2024-03-14

**Authors:** Josef Komenda, Roman Sobotka, Peter J Nixon

**Affiliations:** Center Algatech, Institute of Microbiology of the Czech Academy of Sciences, 37901 Třeboň, Czech Republic; Center Algatech, Institute of Microbiology of the Czech Academy of Sciences, 37901 Třeboň, Czech Republic; Department of Life Sciences, Sir Ernst Chain Building-Wolfson Laboratories, Imperial College London, S. Kensington Campus, London SW7 2AZ, UK

## Abstract

The growth of plants, algae, and cyanobacteria relies on the catalytic activity of the oxygen-evolving PSII complex, which uses solar energy to extract electrons from water to feed into the photosynthetic electron transport chain. PSII is proving to be an excellent system to study how large multi-subunit membrane-protein complexes are assembled in the thylakoid membrane and subsequently repaired in response to photooxidative damage. Here we summarize recent developments in understanding the biogenesis of PSII, with an emphasis on recent insights obtained from biochemical and structural analysis of cyanobacterial PSII assembly/repair intermediates. We also discuss how chlorophyll synthesis is synchronized with protein synthesis and suggest a possible role for PSI in PSII assembly. Special attention is paid to unresolved and controversial issues that could be addressed in future research.

## PSII: a sophisticated solar-driven nano-machine

PSII is one of 2 types of photosynthetic reaction center located in the thylakoid membranes of cyanobacteria, algae, and plants, the other being PSI (Shen 2015). From a functional point of view, PSII is a water:plastoquinone photo-oxidoreductase (EC number 1.10.3.9) catalyzing the light-driven reduction of plastoquinone to plastoquinol and the oxidation of water to molecular oxygen and protons. PSII also contributes to the proton-motive force used to drive ATP synthesis (Qi et al. 2023). Recent advances in structural biology especially cryogenic-electron microscopy (cryo-EM) have provided detailed, high-resolution structures of PSII complexes isolated from a variety of photosynthetic organisms (Cao et al. 2020). These data clearly document the conserved character of the central part of PSII, called the PSII core complex (RCCII).

**Figure 1.**
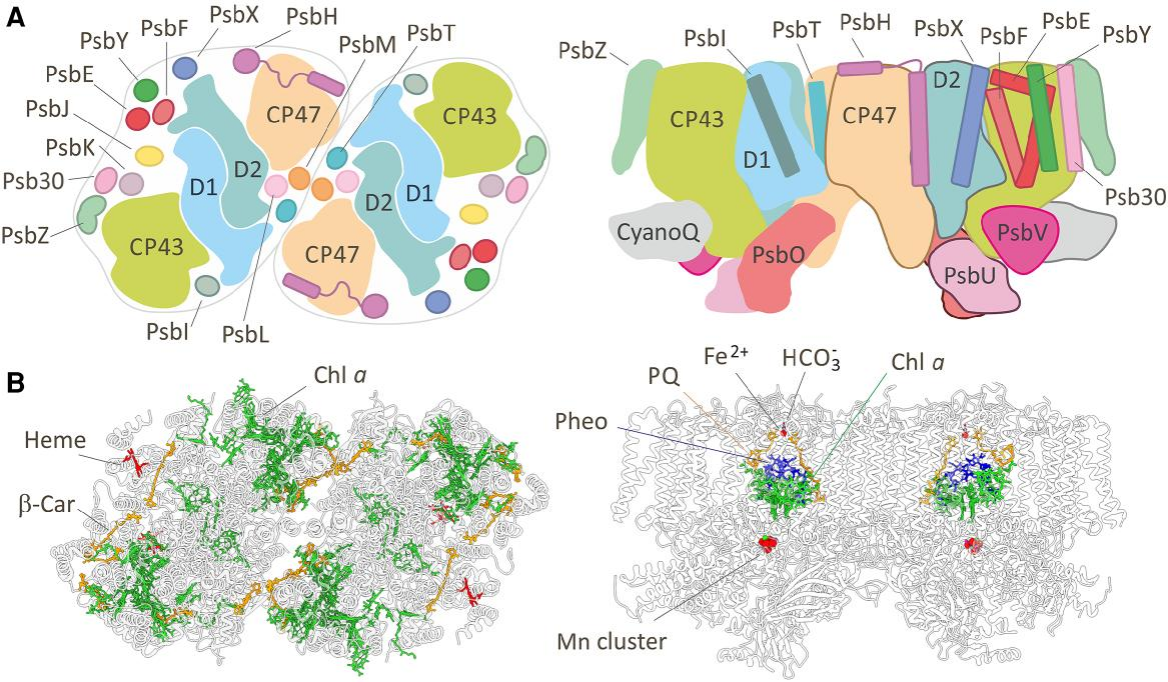
Proteins and cofactors of dimeric oxygen-evolving cyanobacterial PSII. **A)** Cartoon shows all subunits in the available structure of *Synechocystis* PSII (PDB: 7N8O) as a top view from the cytoplasmic side (left) and side-view along the membrane plane (right). **B)** The structure of the same complex highlighting the cofactors. The left side shows the location of chlorophyll *a* (Chl *a*), β-carotene (β-Car), and heme *b*; the right panel shows the plastoquinone (PQ), pheophytin *a* (Pheo), Chl *a*, non-heme iron, bicarbonate, and water-splitting Mn_4_CaO_5_ cluster associated with D1 and D2. Please note that the orientation of the structures in (**B**) corresponds to the cartoons in (**A**).

Efficient assembly of PSII and its prompt repair in response to light-induced irreversible damage, or chronic photoinhibition, is vital for maintenance of PSII activity and growth. Both processes have been intensively studied, especially in cyanobacteria, which have provided the most detailed structural information. Figure 1 presents an overview of the cryo-EM structure of the dimeric oxygen-evolving PSII complex isolated from the cyanobacterium *Synechocystis* PCC 6803 (hereafter *Synechocystis*; Gisriel et al. 2022), which is widely used to study cyanobacterial PSII biogenesis. Each monomer is composed of 4 large and 17 small intrinsic and 4 extrinsic subunits and contains 35 chlorophylls (Chls), 10 β-carotenes, and several other cofactors. The chlorin cofactors involved in the initial steps of light-induced charge separation leading to reduction of plastoquinone and oxidation of water are bound to a central heterodimeric reaction center complex of the D1 and D2 subunits (RCII). The inner antennae, CP47 and CP43, bound symmetrically to D2 and D1, respectively, absorb light energy and deliver it to RCII to drive charge separation. CP43 is also involved with D1 in the ligation of the Mn_4_CaO_5_ metal cluster that oxidizes water (Ferreira et al. 2004; Umena et al. 2011; Shen 2015). These large subunits are surrounded by several smaller transmembrane subunits of less well-defined function.

In contrast to the very conserved core of PSII, different sets of proteins bind to the lumenal side of PSII to optimize the function of the Mn_4_CaO_5_ cluster (Ifuku and Noguchi 2016). *Synechocystis* PSII contains the PsbO, PsbU, PsbV, and PsbQ (also termed CyanoQ) subunits (Fagerlund and Eaton-Rye 2011), whereas in other cyanobacteria CyanoQ is detached during purification and is absent (Michoux et al. 2014). In the case of plant chloroplasts there are 4 extrinsic subunits: PsbO, PsbQ, PsbP, and PsbTn (Wei et al. 2016). The peripheral light-harvesting complexes that associate with PSII to form large PSII supercomplexes are even more diverse (Cao et al. 2020; You et al. 2023). In the case of cyanobacteria, the soluble phycobilisome docks onto the cytoplasmic surface of PSII (You et al. 2023), whereas transmembrane light-harvesting complexes are found in land plants (Lokstein et al. 2021).

## The beginning of the journey: translation of core subunits and formation of modules

**Figure 2.**
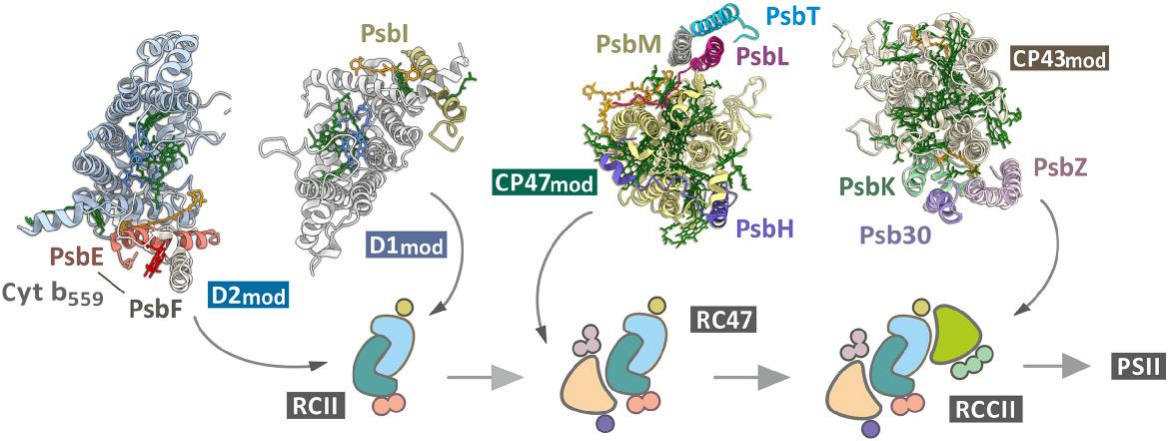
Modular de novo assembly of PSII. According to our model (Komenda et al. 2012a), PSII is built from 4 preassembled modules (D1_mod_, D2_mod_, CP47_mod_ and CP43_mod_) in a step-wise manner. Each module contains 1 core chlorophyll-binding subunit of PSII already associated with a set of small subunits (as indicated) and with pigment cofactors. D1_mod_ and D2_mod_ combine first to produce the RCII reaction center assembly complex, which associates with CP47_mod_ to form the RC47 assembly complex that is then converted to the RCCII non–oxygen-evolving PSII intermediate by binding CP43_mod_. The final steps of assembly to produce oxygen-evolving PSII involve light-driven assembly of the Mn_4_CaO_5_ cluster and attachment of the lumenal subunits and dimerization. The picture was prepared using the cryo-EM structure of *Synechocystis* PSII (PDB: 7N8O) (Gisriel et al. 2022).

PSII assembly proceeds in a step-wise process via discrete assembly intermediates (Nixon et al. 2010; Komenda et al. 2012a; Nickelsen and Rengstl 2013) (Fig. 2). The abundance of these intermediates is generally low in wild type (WT) but can be enhanced in mutants blocked at specific stages of assembly (de Vitry et al. 1989; Komenda et al. 2004) (Fig. 2). Assembly occurs in distinct membrane compartments (biogenesis centers or zones) in cyanobacteria, algae, and plants and is discussed in detail by Nickelsen et al. (this issue).

Although the organization of the PSII biogenesis center remains enigmatic, such a membrane domain should be abundant in the translation and translocation machineries engaged in the production of PSII core subunits. D1, D2, CP43, and CP47 are polytopic membrane proteins that are likely inserted into the membrane via the signal recognition particle (SRP) pathway (Hristou et al. 2019; for review, see Cymer et al. 2015). The cyanobacterial SRP consists of the Ffh protein and a small RNA molecule, which together scan the ribosome exit, recognize hydrophobic sequences, and target them to the SecYEG translocon, a transmembrane channel that allows translocation of nascent protein chains across the membrane. The translocon can associate with the YidC foldase/insertase, which assists the lateral exit of transmembrane helices (TMHs) from the translocon and the translocation of the periplasmic domains across the membrane (for review of YidC, see Hennon et al. 2015). YidC and its plastid homologue ALB3 are involved in the synthesis of all large cyanobacterial Chl-binding proteins of PSII and PSI (Pasch et al. 2005).

Integration of multi-spanning membrane proteins may proceed by either the stepwise integration of single transmembrane segments or by the cooperative insertion of 2 or more TMHs (Ota et al. 1998). However, the precise mechanism for PSII subunits remains to be determined, especially the role of the SecA cytosolic motor (van Wijk et al. 1995; Wang et al. 2017b).

PSII core subunits bind a number of Chl cofactors and, at least in the case of CP43 and CP47, Chl molecules need to be inserted co-translationally as a prerequisite for correct folding (Shen et al. 1993; Manna and Vermaas 1997). The accumulation of D1 and D2 appears less Chl-dependent as unassembled D1 can be detected in *Synechocystis* mutants depleted in Chl (Kiss et al. 2019; Skotnicová et al. 2024) and D2 can be detected in Chl-free etioplasts (Müller and Eichacker 1999). As discussed later, PSII biogenesis needs to be tightly coordinated with Chl biosynthesis and turnover to deal safely with this highly phototoxic pigment. The presence of β-carotene is also critical for the biogenesis of PSII with the stability of the CP47 and CP43 Chl-binding antennae dramatically impaired in a *Synechocystis* mutant lacking this pigment (Sozer et al. 2010). However, in contrast to Chl, carotenoids can safely accumulate as free molecules in the membrane. It is thus expected that β-carotene is transferred spontaneously to apoproteins from a membrane pool during Chl incorporation to provide efficient photoprotection (Domonkos et al. 2013).

The assembly and stability of PSII is also dependent on lipids, which are integral components of PSII (Guskov et al. 2009), with phosphatidyl-glycerol and sulfoquinovosyl-diacylglycerol needed for stabilization of PSII dimers and stable binding of CP43 within PSII (Laczkó-Dobos et al. 2008; Nakajima et al. 2018).

During insertion into the membrane, or soon after, D1, D2, CP47, and CP43 bind pigments and other cofactors and associate with neighboring small membrane PSII subunits to form “assembly modules.” These small, pigmented complexes exist in the membrane autonomously before associating with the other modules to form larger assembly complexes and the final oxygen-evolving PSII complex (Fig. 2). Except for the D1 assembly module (D1_mod_), which probably needs to be produced de novo, the other 3 assembly modules (D2_mod_, CP47_mod_, and CP43_mod_) can be recycled from photodamaged PSII (Komenda and Masojídek 1995; Yao et al. 2012).

Additionally, accessory factors not present in the final functional PSII complex associate transiently with assembly modules and larger assembly intermediates to promote or regulate assembly and protect vulnerable assembly intermediates from damage (Johnson and Pakrasi 2022).

## D1 and D2 assembly modules

Apart from a few exceptions, such as *Euglena*, the D1 subunit is synthesized as a precursor protein (pD1) with a C-terminal extension in the range of 8 to 16 amino-acid residues. This extension is cleaved by the CtpA protease (Anbudurai et al. 1994) to reveal the free carboxyl group of the conserved C-terminal alanine residue of mature D1 (Nixon et al. 1992), which acts as a bidentate ligand to the Mn_4_CaO_5_ cluster (Umena et al. 2011). In cyanobacteria the 16 amino-acid extension is cleaved in 2 steps (Komenda et al. 2007a) via an intermediate form of D1 (iD1) (Inagaki et al. 2001). Whereas pD1 is only detected in an unassembled state, iD1 is present in the D1_mod_ and larger subcomplexes (Komenda et al. 2004).

**Figure 3.**
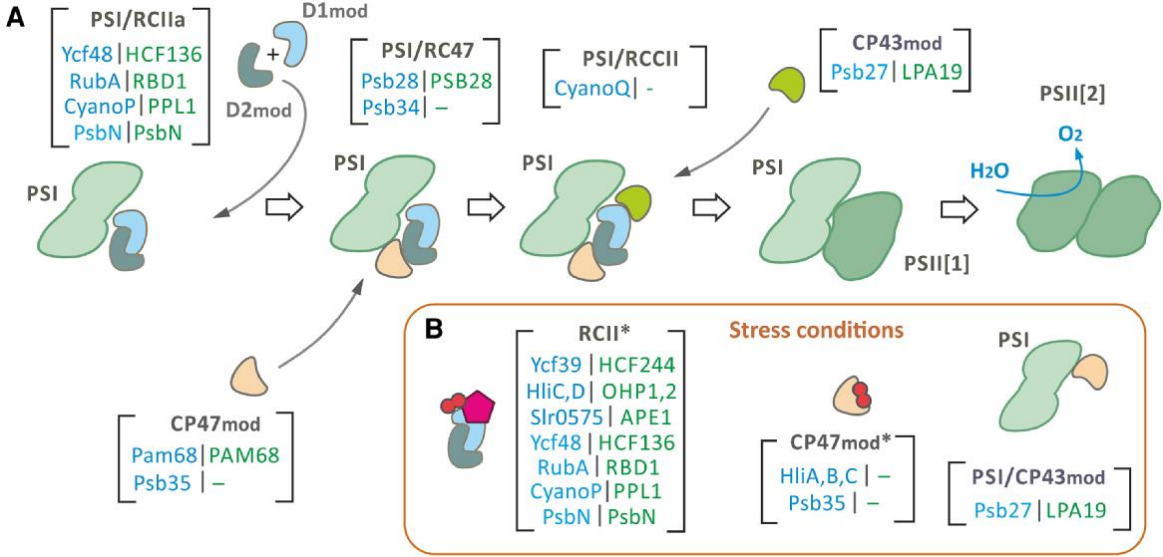
Scheme for involvement of PSI in PSII biogenesis in *Synechocystis*. **A)** Under low-stress conditions, the assembly of PSII begins with the formation of RCIIa reaction center complexes with the assistance of early assembly factors indicated in square brackets. According to our working model (see the text), the PSI monomeric core is either in close vicinity or in physical contact with RCIIa and serves as a scaffold for the later assembly steps. In this scheme, factors involved in each assembly step are listed below the name of the corresponding module or assembly intermediate (blue and green signify cyanobacterial and eukaryotic homologues). It should be noted that some factors are present in multiple assembly complexes (e.g. the RubA rubredoxin-domain protein has been identified in RCIIa, RC47, and RCCII complexes). **B)** Under stress conditions, such as high light, PSI complexes are not produced, and the synthesis of CP47_mod_ and CP43_mod_ is also weakened due to the lack of available Chl. PSII biogenesis is achieved through an alternative pathway that involves the RCII* intermediate, which is photo-protected by the associated Ycf39-HliC/D subcomplex (Ycf39/Hlips). Unlike RCIIa, RCII* exists as an individual entity. The CP47_mod_ and CP43_mod_ antenna mainly originate from disassembled (damaged) PSII. CP47m is photoprotected by HliA/C and HliB/C heterodimers, while free CP43_mod_ can associate with PSI, which serves as a sink for absorbed excitation energy.

The *Synechocystis* D1_mod_ has recently been isolated and found to contain close to stoichiometric amounts of the small PsbI subunit and the Ycf48 assembly factor (Knoppová et al. 2022). The lumenal Ycf48 protein (and its plant homologue HCF136) is important for PSII accumulation, more so in chloroplasts (Meurer et al. 1998; Plücken et al. 2002) than cyanobacteria (Komenda et al. 2008). Ycf48/HCF136 is a 7-bladed beta-propeller protein that acts early in assembly by binding to D1_mod_ and facilitating formation of the RCII complex (Yu et al. 2018) (Fig. 3). Ycf48 also co-purifies with YidC suggesting engagement of Ycf48 with lumenally exposed regions of D1 early in translation, possibly to aid folding and the binding of Chl (Yu et al. 2018).

The isolated *Synechocystis* D2_mod_ contains D2 and cytochrome b_559_ (Cyt b_559_) as anticipated from earlier studies in barley (Müller and Eichacker 1999) and *Synechocystis* (Komenda et al. 2008; Fig. 2). Cyt b_559_ consists of a heme *b* molecule ligated by 2 small proteins [PsbE (alpha) and PsbF (beta)]. Deletion of PsbE or PsbF abolishes PSII assembly (Pakrasi et al. 1988; Morais et al. 1998; Swiatek et al. 2003) by preventing accumulation of D2 (Shukla et al 1992; Komenda et al. 2004). Mutation of the His ligands to the heme profoundly impairs the assembly, photoprotection, and repair of PSII (Morais et al. 2001; Hung et al. 2010; Hamilton et al. 2014), but the precise role of Cyt b_559_ remains enigmatic (Pospíšil 2011).

Also found at substoichiometric levels in D2_mod_ is CyanoP, a protein distantly related to the PsbP extrinsic subunit of chloroplast PSII and the PPL1 subunit (Thornton et al. 2004; Michoux et al. 2010) implicated in PSII assembly in plants (Che et al. 2020). CyanoP appears to protect D2_mod_ from proteolysis, with its absence leading to overaccumulation of newly synthesized D1_mod_ (Knoppová et al. 2016).

Other proteins that co-purify at low levels with D2_mod_ include 2 proteins, Slr0575 and Slr1470, with homologues in chloroplasts (Knoppová et al. 2022). While the function of Slr1470 remains to be determined, Slr0575 has a stabilizing effect on D2 in *Synechocystis* (Knoppová et al. 2022). The plant homologue of Slr0575 called APE1 appears to be important for the acclimation of Arabidopsis to high irradiance (Walters et al. 2003), but its effect on RCII formation in chloroplasts remains unknown. A preliminary report suggests that growth of the *Chlamydomonas ape1* null mutant (Chazaux et al. 2019) is more photosensitive, which agrees with a possible role in RCII formation/stability.

Both the isolated D1_mod_ and D2_mod_ contain Chl and β-carotene but lack Pheo (Knoppová et al. 2022). Given the potential loss of pigment during purification, it remains unclear how many pigments bind to each module in vivo.

## Structure of the photochemically active RCII complex

The RCII complex, formed from the association of D1_mod_ and D2_mod_, has been isolated from a *Synechocystis* strain lacking CP47 (Knoppová et al. 2014; Knoppová et al. 2022) and its cryo-EM structure recently determined (Zhao et al. 2023). Isolated RCII is photochemically active and performs primary charge separation between the P680 electron donor and Pheo electron acceptor (Knoppová et al. 2022). All the expected pigment cofactors of D1/D2 are present in very similar positions to that found in high resolution structures of PSII core complexes (Umena et al. 2011; Wei et al. 2016; Gisriel et al. 2022), except for the loss of 1 β-carotene on the D2 side of the complex, which most probably occurred during purification (Knoppová et al. 2022). Also absent are the plastoquinone electron acceptors, Q_A_ and Q_B_, and the Mn cluster, while the non-heme iron is clearly recognized in the structure. Because it is ligated by 4 histidine residues, 2 provided by D1 and 2 by D2, its insertion most probably occurs during the formation of the heterodimer. The origin of the 2 Pheo molecules in D1/D2 is enigmatic because there is no evidence for the existence of a general, PSII-specific magnesium dechelatase activity essential for generating Pheo *a* from Chl *a*.

The RCII structure also reveals how the Ycf48 assembly factor docks onto the lumenal surface of the complex. In most cyanobacteria the protein is processed at the N terminus, and the resulting N-terminal Cys residue (Cys29 in *Synechocystis*) is lipidated, whereas in chloroplasts lipidation is absent (Knoppová et al. 2021) and the protein contains an additional 19 amino-acid insertion located between blades 3 and 4 (Yu et al. 2018). A conserved arginine-rich patch of Ycf48 interacts with acidic residues on the luminal surface of the D1 protein, while the C-terminal tail of D1 binds into a groove on Ycf48. These regions of D1 ligate the metal ions of the Mn_4_CaO_5_ cluster, and therefore binding of Ycf48 to D1 blocks ligation of Mn ions and formation of the oxygen-evolving cluster. This mechanism avoids photooxidative damage potentially induced by the premature binding of Mn ions to D1 and ensures that assembly of the cluster occurs at the appropriate stage of assembly. Steric clashes between Ycf48 and the lumenal loops of CP47 and CP43 might contribute to detachment of Ycf48 upon formation of larger assembly complexes, although other proteins might participate (Zhao et al. 2023).

Another assembly factor co-purifying with RCII is rubredoxin A (RubA) named after its cytoplasmic rubredoxin domain (Wastl et al. 2000), which is attached to the membrane via a single TMH at its C terminus. In most cyanobacteria the *rubA* gene is located immediately upstream of the *ycf48* gene, suggesting a functional relationship. Indeed, a RubA-Ycf48 fusion protein substitutes for both the RubA and Ycf48 functions, suggesting their proximity in PSII (Kiss et al. 2019). RubA is conserved across oxygenic phototrophs, and a function in PSII was initially suggested by its co-localization with *Guillardia* PSII particles (Wastl et al. 2000). By contrast, RubA in the cyanobacterium *Synechococcus* 7002 was originally proposed to play a role in the biogenesis of Fe-S clusters in PSI (Shen et al. 2002). Later analyses of knockout mutants have confirmed a primary role in PSII function (Calderon et al. 2013; García-Cerdán et al. 2019; Kiss et al. 2019), with the PSI deficiency seen in cyanobacterial mutants an indirect effect (Kiss et al. 2019). Accumulation of a D1 degradation product in a *Chlamydomonas rubA* null mutant (Calderon et al. 2023) implies a role in the correct folding and/or stabilization of D1_mod_. One possibility is that the rubredoxin domain binds ferrous ions and that RubA delivers the non-heme iron to the acceptor side of PSII, thereby stabilizing the D1 structure; however, convincing evidence is still lacking, and a redox role cannot yet be excluded (Kiss et al. 2019; Calderon et al. 2023).

CyanoP, detected in D2_mod_ (Knoppová et al. 2016), remains bound to RCII, although in sub-stoichiometric amounts (Knoppová et al. 2022). CyanoP is apparently much less important for PSII biogenesis than Ycf48 because the CyanoP null mutant behaves like WT. In the absence of D2, CyanoP co-purifies with Ycf48 (Knoppová et al. 2016), indicating an interaction between Ycf48 and CyanoP that may increase the efficiency of RCII formation from D1_mod_ and D2_mod_. Unfortunately, both CyanoP and RubA are absent from the recent RCII cryo-EM structure, and their exact locations within RCII remain unknown (Zhao et al. 2023). Whether PLP1, the closest plant homologue to CyanoP, fulfils a similar role to CyanoP in chloroplasts remains unclear.

Also detected in RCII complexes of *Synechocystis* is PsbN (Knoppová et al. 2022), of unknown function, which had previously been assigned a role in the early steps of PSII assembly in tobacco (Torabi et al. 2014).

## Role of the Ycf39/Hlips complex in photoprotection

The RCII complex (named RCIIa in Knoppová et al. 2022) containing Ycf48, CyanoP, and RubA is detectable in *Synechocystis* grown under standard growth conditions (Fig. 3) and probably represents a cyanobacterial “default” assembly path. Increased irradiance induces the accumulation of a larger form of RCII (designated RCII* in *Synechocystis*), which is associated with binding of an additional pigment/protein complex consisting of the Ycf39 assembly factor (Ermakova-Gerdes and Vermaas 1999; Knoppová et al. 2014) and 2 members of the High-light-inducible protein (Hlip) family termed HliC and HliD (Dolganov et al. 1995; Fig. 3). These Hlip subunits are also designated ScpB and ScpE, respectively, as they are small cab-like proteins (Scps) related to the light-harvesting Chl-*a*/*b*–binding proteins found in chloroplasts (Funk and Vermaas 1999; see below).

The HliC/D pair and 2 other *Synechocystis* Hlips (HliA and HliB) have been shown to bind 4 to 6 Chls and 2 carotenoids in an energy dissipative configuration that leads to the conversion of the absorbed light energy into heat (Staleva et al. 2015; Shukla et al. 2018; Pazderník et al. 2019; Konert et al. 2022). Ycf39 is a member of the atypical short-chain alcohol dehydrogenase/reductase (SDR) family and is of unknown function. Although the Ycf39/Hlips complex is not essential for assembly of PSII, and synthesis of D1 is not markedly affected under low stress conditions, the incorporation of newly synthesized D1 into PSII is affected in the mutant under high light (Knoppová et al. 2014). The HliC/D pair bound to RCII via Ycf39 is therefore likely to play a photoprotective role in dissipating energy absorbed by RCII (Knoppová et al. 2014). Hlips also have the capacity to scavenge free Chls, which would otherwise elicit the generation of singlet oxygen (Sinha et al. 2012) and might participate in Chl recycling (Knoppová et al. 2014), but this role needs further confirmation.

Although progress in understanding the early steps of PSII assembly in plants has been slower in comparison with cyanobacteria, recent evidence suggests that the initial steps are highly conserved. The equivalent complex to Ycf39/Hlips in chloroplasts consists of HCF244 and a pair of Hlip homologues named one-helix protein 1 (OHP1) and one-helix protein 2 (OHP2), which co-purify with RCII and, by analogy, probably play a photoprotective role (Myouga et al. 2018; Li et al. 2019; Maeda et al. 2022). In Arabidopsis all 3 components of the HCF244/OHPs complex are needed for normal D1 synthesis and accumulation of PSII (Link et al. 2012; Chotewutmontri and Barkan 2020; Hey and Grimm 2020), whereas in *Chlamydomonas*, the requirement of OHP2 and HCF244 homologues for D1 synthesis is less strict (Wang et al. 2023).

## CP43-less PSII intermediate (RC47)

The next step in PSII assembly involves the attachment of CP47_mod_ to RCII to form the non-oxygen-evolving RC47 complex (Fig. 2). The binding of CP47_mod_ is rapid, and RCII is hardly detectable in vivo under optimal growth conditions (de Vitry et al. 1989; Komenda et al. 2004).

Analysis of a His-tagged derivative of CP47_mod_ from *Synechocystis* indicates that CP47_mod_ binds a virtually complete set of Chl and β-carotene molecules (Boehm et al. 2011). The conserved Pam68 factor containing 2 TMHs (Armbruster et al. 2010) is thought to act early in the synthesis of CP47 by binding to the apopolypeptide to promote Chl-binding. It was speculated that the coordination of Pam68, YidC, and Ycf48 fixes the nascent CP47 subunit in a position that is amenable to Chl binding (Bučinská et al. 2018). In chloroplasts PAM68 acts in concert with the DEAP2 factor, with the lack of both proteins resulting in loss of functional PSII (Keller et al. 2023).

CP47_mod_ contains several neighboring small transmembrane subunits: PsbH, PsbL, PsbM, and PsbT (Boehm et al. 2011). PsbM and PsbT form part of the interface between the PSII monomers within the dimer (Umena et al. 2011) and contribute to the formation of the PSII dimer (Bentley et al. 2008). Loss of either PsbL or PsbT has multiple effects, including modification of the properties of Q_A_ and Q_B_ and enhanced sensitivity to photodamage (Luo et al 2014; Fagerlund et al. 2020). The single TMH of PsbH interacts with helices II and III of CP47, and its N-terminal tail is folded over the cytoplasmic (stromal) surface of CP47. PsbH helps bind Chl and β-carotene and its binding to CP47 might help detach Pam68 (Bučinská et al. 2018).

RC47 is relatively abundant in oxygenic phototrophs and represents a heterogeneous mixture of complexes formed during both assembly and repair of PSII (Adir et al. 1990; Barbato et al. 1992; Komenda and Masojídek 1995; Boehm et al. 2012b). Since CP43 provides a Glu ligand to the Mn_4_CaO_5_ cluster (Ferreira et al. 2004), RC47 assembly complexes lack the Mn_4_CaO_5_ cluster but are still capable of light-driven electron transfer from tyrosine Y_z_ to Q_A_ (Rögner et al. 1991; Boehm et al. 2012b). A subpopulation of RC47 containing the early assembly factors Ycf48, RubA, and CyanoP has also been detected and may represent PSII in the process of repair (Knoppová et al. 2016; Yu et al. 2018; Kiss et al. 2019).

The Psb28 subunit is present in both RC47 and in larger core complexes containing CP43 (RCCII) (Kashino et al. 2002; Dobáková et al. 2009; Sakata et al. 2013; Bečková et al. 2017a) (Fig. 3). Early crosslinking experiments proposed that the extrinsic Psb28 subunit was in contact with the cytoplasmic N-terminal tails of both Cyt b_559_ subunits (Weisz et al. 2017). However, the latest cryo-EM structures have revealed that D1, D2, and CP47 are the main interacting partners (Xiao et al. 2021; Zabret et al. 2021). Binding of Psb28 induces substantial structural changes to the cytoplasmic regions of D1 and D2 so that the Q_B_ pocket is distorted, the non-heme iron is ligated by residue Glu241 in the D2 subunit, rather than by bicarbonate in oxygen-evolving PSII, and binding of CP43_mod_ is destabilized.

These substantial conformational changes may stabilize reduced Q_A_ and protect PSII from photodamage by reducing the production of singlet oxygen from Chl triplet states produced via charge recombination (Brinkert et al. 2016; Zabret et al. 2021). Furthermore, attachment of Psb28 to the cytoplasmic surface of RC47 aids photoprotection by preventing docking of the phycobilisome. Nevertheless, the physiological function of Psb28 is still not yet clear. Levels of RC47 are almost undetectable in a *psb28* null mutant of *Synechocystis* and assembly as well as repair seem to proceed faster than in WT (Dobáková et al. 2009, Bečková et al. 2017a). Binding of Psb28 therefore seems to block assembly of a fraction of newly assembled PSII at the stage of RC47 (or RCCII with weakly bound CP43), perhaps to confer a special function to a subpopulation of RC47, such as in Chl biosynthesis (see below).

In *Synechocystis* 2 Hlip heterodimers (HliA/C and HliB/C) associate with CP47_mod_ during stress (Fig. 3) and are detected in RC47 as well as in the PSII core complex (RCCII) (Promnares et al. 2006; Yao et al. 2007; Konert et al. 2022). These Hlips are likely to photoprotect PSII assembly intermediates containing CP47, but definitive evidence is lacking. Cyanobacteria also contain a protein, designated Psb34, with a long N-terminal tail similar in primary structure to the N-termini of HliA/HliB but lacking the Chl-binding domain. Cryo-EM structures have revealed that N-terminal part of Psb34 binds to the cytoplasmic side of RC47 and RCCII in the vicinity of Psb28 (Xiao et al. 2021; Zabret et al. 2021). It has been suggested that Psb34 binds to the same binding site as Hlip heterodimers and so promotes detachment of Hlip heterodimers during the later stages of PSII formation (Rahimzadeh-Karvansara et al. 2022). In contrast the recently discovered Psb35 subunit binds to CP47_mod_ and other PSII assembly intermediates containing CP47_mod_, helping to stabilize the binding of Hlips and increase the stability of these complexes in the dark (Pascual-Aznar et al. 2021).

## Building the PSII core (RCCII)

Attachment of CP43_mod_ to RC47 forms the RCCII complex. Free CP43_mod_, which consists of CP43 and small PSII subunits PsbZ, PsbK, and Psb30 (Boehm et al. 2011; Komenda et al. 2012a; Fig. 2), is relatively abundant in membranes (Vermaas et al. 1988; Komenda et al. 2004) and, like CP47_mod_, probably contains its full complement of Chl and carotenoid cofactors (Boehm et al. 2011). The PsbK subunit is needed for stable attachment of CP43_mod_ to RC47 (Komenda et al. 2012b), but the role of PsbZ is unclear (Bishop et al. 2007); however, the tobacco PsbZ null mutant shows a lower level of PSII-LHCII supercomplex (Swiatek et al. 2001). The small PsbJ subunit (Fig. 1) has not been detected in the isolated CP43_mod_ or RC47 complexes and might bind to RCCII late in assembly (Choo et al. 2022).

The CP43_mod_ also associates with the Psb27 assembly factor (Nowaczyk et al. 2006), which binds to the large lumenal loop of CP43 (loop E) interconnecting TMHs 5 and 6 (Liu et al. 2011; Komenda et al. 2012b) and assists its attachment to RC47 (Komenda et al. 2012b). Psb27 folds into a robust 4-helix bundle (Cormann et al. 2009; Mabbitt et al. 2009; Michoux et al. 2012; Xingxing et al. 2018), which may protect PSII from lumenal proteases (Komenda et al. 2012b).

Psb27 is associated with isolated PSII complexes that lack a functional Mn cluster (Nowaczyk et al. 2006; Roose and Pakrasi 2008). Recent cryo-EM structures suggest that binding of Psb27 to CP43 may impede binding of PsbO to maintain diffusional access of Ca^2+^ and Mn^2+^ ions into PSII to enable assembly of the Mn cluster (Huang et al. 2021, Zabret et al. 2021). However, there is little difference in the structures of inactive PSII with and without bound Psb27 (Huang et al. 2021; Zhao 2023), so the role of Psb27 may be to inhibit assembly of the Mn cluster by constraining the conformational flexibility of the large lumenal loop of CP43 (Avramov et al. 2020; Tokano et al. 2020). Psb27 thus could stabilize a pool of “back-up” PSII complexes in the membrane that can be rapidly photoactivated following detachment of Psb27 (Komenda et al. 2012b), possibly driven by binding of CyanoQ, whose binding site overlaps that of Psb27 (Gisriel and Brudvig 2022). Recent ideas also suggest that Psb27-containing complexes are subject to non-photochemical quenching of excitation energy to help prevent photodamage (Johnson et al. 2022).

Psb27 (Nowaczyk et al. 2006), like CyanoP (Ishikawa et al. 2005), Ycf48 (Knoppová et al. 2021), and CyanoQ/PsbQ (Fagerlund and Eaton-Rye 2011), is a lipoprotein, which might help anchor PSII complexes in biogenesis regions via its lipid moiety, whereas the chloroplast homologues are not lipidated which may be related to differences in how and where PSII is assembled.

Although the binding sites and precise roles of Psb27 in chloroplasts remain to be determined, a tobacco Psb27 homologue was recently detected in a novel monomeric PSII assembly intermediate containing the PSBS subunit (Fantuzzi et al. 2023), and Arabidopsis homologues have been implicated in PSII repair (Psb27-1, Chen et al. 2006) and the maturation of D1 (Psb27-2, Wei et al. 2010).

## Assembly of the Mn cluster

Formation of oxygen-evolving PSII involves light-driven assembly of the Mn_4_CaO_5_ cluster (in a process termed photoactivation) and subsequent attachment of the lumenal extrinsic subunits, which shield the active cluster. Early studies in *Synechocystis* suggested that the tetratricopeptide PratA protein present in the periplasm was involved in pre-loading D1 with Mn early in assembly before the RCCII is formed (Stengel et al. 2012). However, this now seems unlikely given the recent structure of the Ycf48-binding site in PSII, which prevents binding of Mn to mature and precursor forms of D1 (Zhao et al. 2023). It is therefore more likely that Mn binds to PSII later in the assembly process after release of Ycf48 and after formation of RCCII, when the full complement of amino-acid ligands to the cluster are available.

The photoactivation process is known to consist of light-dependent and light-independent events (Bao and Burnap 2016), but the molecular details remain unclear. The first step is the light-driven oxidation of a single Mn^2+^ ion bound at a high-affinity binding site within D1 (Diner and Nixon 1992; Nixon and Diner 1992) close to Y_z_, the immediate oxidant of the cluster. Recent cryo-EM structures of PSII complexes lacking the intact cluster have provided hints on the location of this Mn^2+^ ion in both cyanobacterial (Zabret et al. 2021) and plant complexes (Graça et al. 2021). The C-terminal tails of D1 and D2 are difficult to model in PSII structures lacking the Mn cluster, suggesting a high degree of flexibility (Gisriel et al 2020; Huang et al. 2021; Zabret et al. 2021; Zhao et al. 2023). It is likely that assembly of the Mn cluster is coupled to local conformational changes in D1 so that residues in the D1 tail and CP43 correctly ligate the cluster. These structural changes then trigger reorientation of the D2 C-terminal tail and the lumenal loop of CP43 to permit binding of PsbO and the other extrinsic proteins to the lumenal surface of PSII to bind and seal off the Mn cluster (Zhao 2023).

Cyanobacterial oxygen-evolving PSII exists in the form of both a dimer and monomer, although the isolated dimer is more active than the monomer (Nowaczyk et al. 2006). It has been assumed that the oxygen-evolving PSII dimer is assembled from active PSII monomers. However, recent cryo-EM analyses have revealed that isolated dimeric PSII complexes are heterogeneous (Huang et al. 2021; Lambertz et al. 2023; Zhao 2023). Besides fully assembled active dimers, PSII complexes can be found as inactive dimers and semi-active dimers with just 1 of the 2 complexes containing an assembled cluster (Zhao 2023), and dimers with Psb27 bound to 1 or both complexes (Lambertz et al. 2023). Thus, assembly of the Mn cluster appears to occur by parallel pathways involving both monomeric and dimeric complexes.

## PSII is a mosaic of new and recycled components formed during cycles of disassembly and assembly

PSII is a weak link in photosynthesis due to its vulnerability to light-induced irreversible damage caused by the production of reactive oxygen species (ROS) in PSII and by the reactivity of highly oxidizing species needed to drive the oxidation of water (Diner and Rappaport 2002; Pospíšil 2009). Photodamage is an inevitable intrinsic feature of the complex and several “donor” and “acceptor” side mechanisms have been proposed depending on the site of primary impairment (for review, see Vass 2012). Although there is no general agreement on which mechanism prevails in nature, all involve formation of highly oxidizing species that irreversibly damage protein and co-factors within PSII.

Specific adaptations have been discovered that increase the intrinsic resistance of PSII from damage. These include synthesis of a specific form of cyanobacterial D1 in high light that contains a Glu residue rather than a Gln in the vicinity of the photoactive Pheo that reduces the production of singlet oxygen via charge recombination (Vass 2011). In the case of the extremely light-resistant alga *Chlorella ohadii*, an additional protein binds close to the Q_B_-binding site in oxygen-evolving PSII, possibly to reduce oxidative damage (Fadeeva et al. 2023). Whether expression of this protein will confer photoprotection in other organisms is unknown.

**Figure 4.**
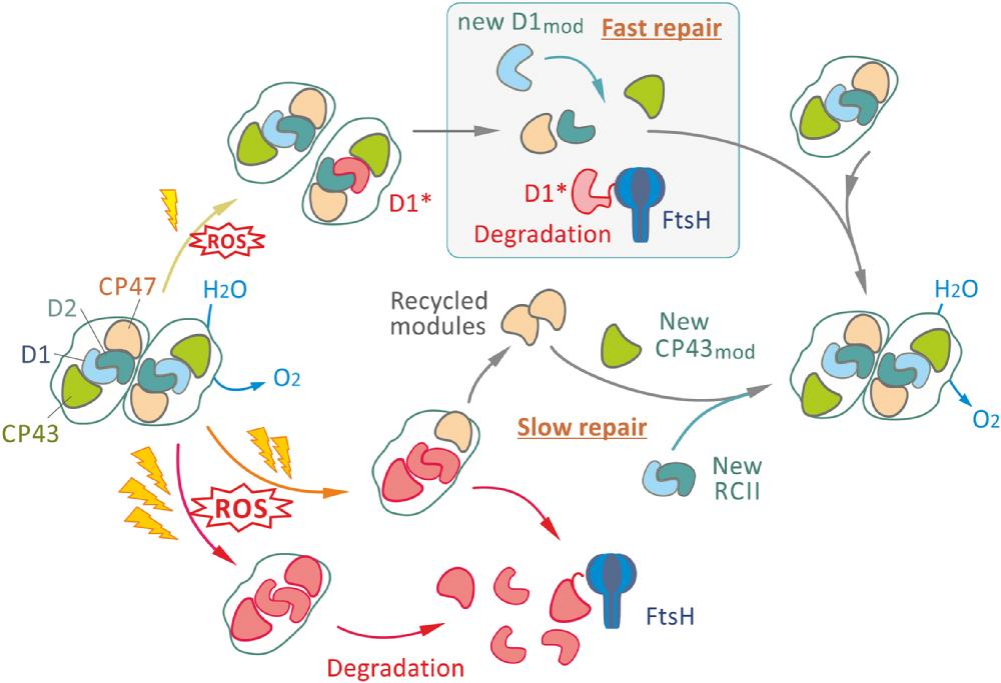
Scheme for PSII repair. The dimeric PSII inactivated by light is partially disassembled and can be promptly repaired by detachment of CP43_mod_, fast FtsH-mediated D1 degradation, D1 synthesis, and reassembly of the active dimeric PSII (Fast repair, upper box). Under harsh oxidative stress when fast repair cannot cope with the high rate of oxidative inactivation, other PSII subunits are also oxidized by ROS and their replacement and reassembly into the active dimeric PSII is also needed (Slow repair, lower part of the scheme).

Once damaged, rather than resynthesize PSII de novo, complexes are “repaired” by selectively degrading the damaged subunit, replacing it by a newly synthesized subunit and recycling the undamaged components (Fig. 4). D1 is preferentially inactivated because it binds most of the co-factors that cause damage through oxidative side-reactions (Ferreira et al. 2004) and is the subunit that is replaced most often during so-called rapid D1 turnover. Under stress conditions, not only D1 but also D2, CP43, and finally CP47 are irreversibly damaged and replaced (Komenda and Masojídek 1995; Jansen et al. 1999; Yao et al. 2012). Thus, it is now clear that PSII repair is a much broader concept and can include the replacement of 1 (D1), 2 (D1, D2), 3 (D1, D2 and CP43), or more PSII subunits while the remaining “undamaged” subunits are recycled (see Fig. 4).

As D1 lies at the heart of the PSII complex, replacement requires partial detachment of the CP43_mod_ to allow the fast degradation of the damaged D1 copy and insertion of a new version. Although replacement of D1 is widely assumed to take place in the RC47 complex, it cannot be totally excluded that damaged PSII disassembles into the individual modules (CP47_mod_, D2_mod_, CP43_mod_, and damaged D1_mod_) and then rapidly reassembled using a newly synthesized D1_mod_ similar to that seen for de novo assembly. The recent suggestion that PSII repair may involve a novel CP47_mod_/CP43_mod_ complex lacking the RCII complex (Weisz et al. 2019) remains highly controversial (Bečková et al. 2022).

Since the incorporation of newly synthesized D1 into RC47 is extremely fast, the co-translational incorporation of D1 into RC47 was originally proposed (Zhang et al. 1999). Given the known structure of the heterodimeric D1/D2 complex (Fig. 1) and assuming sequential insertion of D1 helices into the membrane, only the last 2 TMHs (IV and V) of D1 (Fig. 1) will potentially interact co-translationally with the corresponding helices of D2.

Currently it is far from clear what triggers disassembly of damaged PSII. Early studies using isolated chloroplast thylakoids (Adir et al. 1990; Barbato et al. 1992) or cyanobacterial cells with inhibited protein synthesis (Komenda and Masojídek 1995) suggested that monomerization of dimeric PSII preceded D1 degradation (Fig. 4). The interface between PSII monomers contains a belt of lipid molecules that is important for dimer stability (Guskov et al. 2009). Activation of a lipase, which would cleave these lipids, could potentially induce monomerization and indeed a PSII-associated lipase, LipA, has recently been implicated in D1 degradation in *Synechocystis* (Jimbo and Wada 2023). This lipase could also be involved in the detachment of CP43, since a lipid layer is also located between CP43 and D1 within PSII (Guskov et al. 2009). In addition, oxidative damage to PSII, such as to a bound cofactor, lipid or protein side-chain damage, may weaken the binding of CP43_mod_ to RC47. Indeed, recent work has identified an important role for an oxidized Trp residue in the N-terminal tail of D1 in the detachment of CP43 (Kato et al. 2023).

On the other hand, additional auxiliary proteins may interact with RCCII to facilitate controlled disassembly and repair and so minimize the production of ROS from damaged complexes, which would otherwise inhibit protein synthesis (Nishiyama et al. 2001). This is especially important in eukaryotes, where damaged complexes migrate laterally in membranes to sites of protein synthesis/degradation (Puthiyaveetil et al. 2014). A possible *Chlamydomonas* PSII “repair” complex has recently been isolated and characterized by cryo-EM (Liu et al. 2023). The complex contains 3 additional, previously unidentified protein factors that participate in the detachment of PsbO and the CP29 antenna and blockage of the Q_B_ site. In chloroplasts, the phosphorylation of PSII core subunits (CP43, D2, D1, PsbH) is widely assumed to regulate PSII disassembly. However, these conclusions have relied heavily on the analysis of kinase and phosphatase knockout mutants that may have impacts outside PSII (Longoni and Goldschmidt-Clermont 2021).

## Proteases involved in PSII repair

The main players involved in degrading damaged PSII subunits belong to the FtsH family of ATP-dependent membrane-embedded metalloproteases (Yi et al. 2022). *Synechocystis*, like most cyanobacteria, encodes 4 different FtsH subunits, with a heterohexameric complex of SynFtsH2 and SynFtsH3 (the FtsH2/3 complex) responsible for selective degradation of D1 (Silva et al. 2003; Boehm et al. 2012a) and for removal of mutated proteins and assembly intermediates (Komenda et al. 2006). For D1, the proposed mechanism involves recognition of the N-terminal tail of damaged D1 by the FtsH protease complex and subsequent processive degradation (Nixon et al. 2005; Komenda et al. 2007b).

From a phylogenetic viewpoint, FtsH2 (a type-B subunit) and FtsH3 (a type-A subunit) emerged early in the evolution of oxygenic photosynthesis (Shao et al. 2018). The corresponding homologues in algae and plants appear to fulfil the same role as SynFtsH2/3 (Sakamoto et al. 2003; Malnoë et al. 2014). The FtsH2/3 complex also co-purifies with preparations of D2_mod_ and RCII from *Synechocystis* (Knoppová et al. 2022), which supports a role in the degradation of both D1 and D2 within RCII (Krynická et al. 2015). The FtsH2/3 complex also interacts with prohibitin, a member of the band 7 protein family, which might regulate FtsH activity (Boehm et al. 2009; Boehm et al. 2012a).

Accumulation of the FtsH2/3 protease complex in *Synechocystis* is dependent on the Psb29 subunit, which was originally identified as a PSII assembly factor (Kashino et al. 2002). Psb29 interacts directly with FtsH, but it remains unclear whether it is involved in assembly or stabilization of FtsH complexes (Bečková et al. 2017b). The plant homologue of Psb29, termed Thylakoid Formation 1, THF1 (Keren et al. 2005), is expected to play a similar role in chloroplasts (Bečková et al. 2017b). The *Chlamydomonas* CrFtsH1/2 complex exhibits increased turnover in the light (Wang et al. 2017a), with accumulation dependent on the peptidyl-prolyl cis-trans isomerization activity of immunophilin CYN28 (Fu et al. 2023); in *Arabidopsis*, FtsH turnover requires the EngA GTPase, which suggests a possible role for phosphorylation in FtsH action (Kato et al. 2018).

By contrast, the homo-oligomeric SynFtsH4 complex, which is also found in *Synechocystis* thylakoids, is not involved in the degradation of damaged PSII subunits; rather, it controls the level of Hlips and possibly other PSII assembly factors (Krynická et al. 2023). What determines the substrate specificity of FtsH complexes remains unknown.

Selective D1 degradation in cyanobacteria is synchronized with the synthesis of D1 so that when the D1 subunit is not available the degradation of damaged D1 is postponed and is closely followed by degradation of D2 (Komenda and Barber 1995; Mulo et al. 1997; Komenda et al. 2000; Masuda et al. 2023). The mechanism for this synchronization is unclear but might reflect the degradation of damaged D1 by FtsH attached to the Sec translocon (Yu et al. 2018).

The second group of proteases involved in the degradation of PSII components, especially D1, in chloroplasts are the Deg serine proteases, which are located in both the stroma and lumen and are considered as back-up proteases that cleave exposed loops of D1 and, possibly, other proteins to enhance FtsH-mediated degradation (Kato and Sakamoto 2009). Although important for conferring resistance to light stress, the *Synechocystis* Deg proteases do not play an important role in D1 degradation (Barker et al. 2006).

## Chl biosynthesis and delivery to PSII during biogenesis

As already noted, unbound Chl is phototoxic and synthesis of Chl-binding proteins needs to be strictly synchronized with Chl biosynthesis and organized in a way to prevent leakage of Chl molecules into the membrane. The terminal enzyme of the Chl biosynthesis pathway, Chl synthase, is associated with the YidC insertase in cyanobacteria (Chidgey et al. 2014) and possibly in plants (Proctor et al. 2018), which has led to speculation that the Chl biosynthesis enzymes are organized close to the translocon (Sobotka 2014) and that newly formed Chl passes directly from the Chl synthase to the nascent polypeptide chain (Chidgey et al. 2014) in a co-translational mechanism, as suggested from early studies (Eichacker et al. 1990; Mullet et al. 1990). However, some Chls are now known to be bound at the interface of two subunits in photosynthetic complexes suggesting some post-translational binding of Chl. Also, analysis of purified D1_mod_ and D2_mod_ indicates that at least some Chl molecules are inserted after the folding and formation of modules (Knoppová et al. 2022). Nonetheless, it is likely that this post-translational Chl insertion still occurs in close vicinity of the translocon (and Chl synthase), where a limited pool of free Chl might be present. Its toxicity could be mitigated by Hlips and a high concentration of carotenoids. The stable assembly of D1 and D2 into the RCII complex requires Chl (Knoppová et al. 2022) with insertion facilitated by Ycf48 (Yu et al. 2018). The fact that this assembly factor can be co-isolated with YidC (Yu et al. 2018) supports the preloading of D1_mod_ and D2_mod_ with pigments on the periphery of the translocon.

Analysis of a wide range of PSII assembly mutants suggests that Chl biosynthesis is dependent on the ongoing assembly of PSII in cyanobacteria (Bečková et al. 2017a; Yu et al. 2018; Kiss et al. 2019) and chloroplasts (Plücken et al. 2002; García-Cerdán et al. 2019; Che et al. 2022). These mutants are PSI deficient, which appears to be a consequence of such regulation. Possibly, PSII assembly intermediates may interact with Chl biosynthesis enzymes, stabilize them and channel new Chl into newly synthesized Chl-binding proteins. This hypothesis is supported by detection of Mg-protoporphyrin IX monomethylester cyclase in RC47 and/or RCCII assembly intermediates containing Psb28 (Dobáková et al. 2009) but needs further support.

Importantly, the lifetime of Chl exceeds that of individual PSII Chl-binding proteins, and hence Chl is recycled (Yao et al. 2012). As yet, the Chl-binding proteins involved in this process remain unknown. D1, D2, and partly CP43 are synthesized in *Synechocystis* even when de novo Chl biosynthesis is inhibited, suggesting that they can utilize previously synthesized Chl molecules released from other Chl-binding proteins (Kopečná et al. 2013; Hollingshead et al. 2016). By contrast, synthesis of CP47 and formation of PSI trimers are dependent on the sufficient supply of new Chl occurring under optimal growth conditions when fast cell proliferation occurs (Kopečná et al. 2012). This would indicate that PSI monomers need additional Chls to trimerize, and that this Chl could then be released upon monomerization.

## A possible role for PSI in PSII biogenesis

The recent co-isolation of PSII assembly modules and assembly intermediates with PSI complexes has provided biochemical evidence for the possible involvement of PSI in PSII biogenesis (Fig. 3). Affinity purification of various PSII assembly intermediates and PSI complexes has led to the isolation of CP43_mod_ (Fig. 3; Komenda et al. 2012b; Kopečná et al. 2015; Strašková et al. 2018) and RC47 (Kiss et al. 2019; Pascual-Aznar et al. 2021) complexes bound to monomeric PSI complexes as well as CP47_mod_ and RCCII bound to trimeric PSI (Bečková et al. 2017a; Pascual-Aznar et al. 2021). Although more work is needed to confirm the structure and physiological relevance of these complexes (Zhao et al. 2023), these results suggest that the biogenesis pathways of PSI and PSII may be intertwined (Fig. 3). This is further supported by findings that some assembly factors like Psb27 (Komenda et al. 2012b), Ycf48 (Yu et al. 2018), and Psb35 (Pascual-Aznar et al. 2021) seem to be shared by both PSI and PSII. PSI may therefore play a role as a scaffold that transiently binds PSII assembly modules and intermediates to supply them with its own weakly bound Chl molecules and photoprotect them by dissipating excess light energy via excitation energy transfer (or “spill-over”) to PSI (Bečková et al. 2017a; Strašková et al. 2018; Fig. 3).

## Future outlook

Thanks to the dramatic progress in structural biology, especially cryo-EM, we are entering an era where structures of low-abundance complexes involved in PSII assembly and repair can be rapidly determined. A combination of affinity purification of tagged complexes from WT and mutants and cryo-EM will allow us to address the binding sites and possible functions of the many accessory factors so far implicated in PSII biogenesis (see Johnson and Pakrasi 2022 for recent list). Some of these are specific for cyanobacteria or chloroplasts and probably reflect specific adaptations.

Key questions to address in the future include how Chl is handled during the synthesis of Chl-binding proteins and during degradation of damaged PSII subunits and how the Mn_4_CaO_5_ cluster is assembled in PSII during the process of photoactivation. In terms of PSII repair, cryo-EM analysis combined with mass spectrometry of PSII complexes isolated from cells at various stages of photodamage will reveal insights into the modifications and accompanying structural changes that occur to trigger selective D1 degradation. So far work in this area has been restricted to identifying sites of damage in isolated PSII complexes (Kale et al. 2017). Ultimately, it might even be possible to obtain snapshots of PSII complexes undergoing assembly/repair in situ using advanced cryo-tomography techniques as recently applied in the red alga *Porphyridium purpureum* (You et al. 2023) to study PSI/PSII megacomplexes. Ultimately, fundamental knowledge in this area might be exploited to improve the efficiency of PSII assembly and repair, especially under abiotic stress conditions, to increase biomass yields.

## Data Availability

No new data were generated or analysed in support of this research.

## Dive Curated Terms

The following phenotypic, genotypic, and functional terms are of significance to the work described in this paper:

OHP2 Gramene: AT1G34000
OHP2 Araport: AT1G34000

ALB3 Gramene: AT2G28800
ALB3 Araport: AT2G28800

APE1 Gramene: AT5G38660
APE1 Araport: AT5G38660

HCF136 Gramene: AT5G23120
HCF136 Araport: AT5G23120

PLP1 Gramene: AT4G37070
PLP1 Araport: AT4G37070

Psb28 Gramene: Sll1398
Psb28 Araport: Sll1398
